# Chemical compounds released by combustion of polymer composites flat belts

**DOI:** 10.1038/s41598-021-87634-9

**Published:** 2021-04-15

**Authors:** Piotr Krawiec, Łukasz Warguła, Dorota Czarnecka-Komorowska, Paweł Janik, Anna Dziechciarz, Piotr Kaczmarzyk

**Affiliations:** 1 Polymer Processing Division, Institute of Machine Design, Faculty of Mechanical Engineering, Poznan University of Technology, 60-965 Poznań, Poland; 2 Scientific and Research Centre for Fire Protection, National Research Institute, 05-420 Józefów, Poland

**Keywords:** Techniques and instrumentation, Engineering

## Abstract

Machines and devices for the production, transport and segregation of products are placed in production and storage rooms. Flat conveyor and drive belts are very often used for their construction. Due to heavy loads and difficult operating conditions, these belts can catch fire and, as a result, become the main source of air contaminants harmful to human health and life. This article examines the emission level of toxic chemical compounds most often produced during the thermal decomposition and combustion of flat drive and conveyor belts. Six types of flat belts, which were made of various polymer materials, i.e., polyamide, rubber, and polyurethane, and were pyrolyzed in a tube furnace at 950 °C, were tested for emission. Using an Fourier transform infrared spectroscopy gas analyser, five gaseous products of combustion were identified, i.e., carbon mono oxide, carbon dioxide, hydrogen cyanide, hydrogen bromide and sulfur dioxide (SO_2_). Chemical analysis showed that SO_2_ compounds and hydrogen bromide were present in only two samples. The test results indicate that gas emission concentration limits for all the tested belts were significantly exceeded. A comparative analysis of the concentration limits of V-belts described in the authors' earlier works shows that flat belts demonstrate lower emission levels of harmful compounds than V-belts. In addition, research has shown that compared to traditional rubber-based belts, belts made of modern materials exhibit no emission of hydrogen chloride compounds during thermal decomposition and combustion.

## Introduction

Some of the early examples of bands used in drive transmission technology included hemp ropes (a round belt) and leather (a flat belt). The emergence of these two types of belts was associated with the availability and low cost of the acquired materials. Both ropes and leather were impregnated and preserved with various types of natural products, such as resins, oils, and tar. The advanced technique of curing and gluing leather became the basis for the production of high-quality belts, which prevailed in this form until the rubber vulcanization technique was mastered. Flat belts are used in machines and devices, performing both driving^1^ and transport^2,3^ functions. Classic and commonly used belts are made of fabric-rubber composites reinforced with cord^4,5^. Currently, thanks to the development of construction materials, these belts are produced from various multilayer polymeric materials, e.g., poly(ethylene terephthalate), polyamide, polyurethane and polyoxymethylene^6,7^. Specialist literature provides the test results of the mechanical properties, which also examine the effect of the machining (perforation)^8^ of flat belts. However, there is little up-to-date information regarding the effects of high temperature on this type of band and its resistance to combustion. During the transmission operation, the belts are exposed to high temperature as a result of damage to machine components or external factors^9–11^. For example, there may be a seizure and stoppage of the snub pulleys or intermediate pulleys, which change the nature of the interaction between the pulley and the belt from rolling to sliding friction^12^. Mechanical damage to machine components^13,14^, transmission contamination^15^ or external factors, such as prolonged exposure to high temperature^16^, may also be the causes of belt combustion. Currently, fibre-reinforced polymer composites are commonly used in drive and conveyor belts. Such materials not only provide an appropriate strength-to-weight ratio of the belt but also demonstrate positive properties such as a high resistance to pulling and bending; stiffness and vibration damping; and resistance to corrosion, wear, impact load and high temperature^17^. Due to this wide range of features, composite materials are now used in electromechanical, construction, aviation, automotive, biomedical, and marine applications and many other areas of production.

According to the literature reviewed by the authors, composites of these materials have not been examined so far in terms of the emission of chemical compounds during thermal decomposition and combustion. Only V-belts, which are mainly used as transmission bands, not as conveyor belts, have been tested. Research has shown that these belts pose a serious threat to human life and health during a fire^10,11^. Therefore, it is necessary to try and use materials with relatively low impact on the environment to maintain the standards for the emission of harmful compounds described in documents and scientific works on fire protection^17,18^. The literature^19–24^ also describes the basic effects of the contact of plastics with fire, i.e., high temperature, smoke and toxic product emission during the thermal decomposition and combustion of materials, oxygen deficiency, and damage to structural elements. It has been proven that carbon black powerfully adsorbs toxic gases, accelerating their absorption by the human body and the natural environment^25,26^.

The authors of the test results have jointly determined the flammability of rubber materials, which are common to conveyor tyres, conveyor belts and insulation of power cables, and have compared the thermal magnitude of the cargo quantities of these materials to other fuels that are publicly transported^27,28^. In turn, the smoke production of a material and the toxicity of the products of its thermal decomposition and combustion depend mainly on the chemical composition and combustion temperature of the materials.

The aim of this study was to evaluate the chemical composition of gases emitted during the combustion of belts, which have a direct impact on the level of emissions of chemical compounds harmful to human health.

## Experimental

### Materials

The tests covered belts are made of several layers of materials, including fibres, fabrics, most often plastics, i.e., thermoplastic polyurethane (TPU), polyamide (PA) and acrylonitrile butadiene rubber (NBR), and in one case, LL2 natural leather.

Six types of commercial belts, NBR/PA fabric/PA film/PA6/soft NBR (abbreviation XH), NBR/TPU/PES fabric/TPU/NBR (abbreviation TLA), thermoplastic connection (abbreviation TC), NBR/PA fabric/PA film/PA fabric/NBR (abbreviation SG), and NBR/PA film/special fabric (abbreviation KSG), serving both the drive and transport functions were used for the tests, including five manufactured by NITTA Co. (Osaka, Japan)^29^ and one of them leather/PA/leather (abbreviation LL2) by Chiorino* (Biella, Italy)^30^.

**Figure 1:**
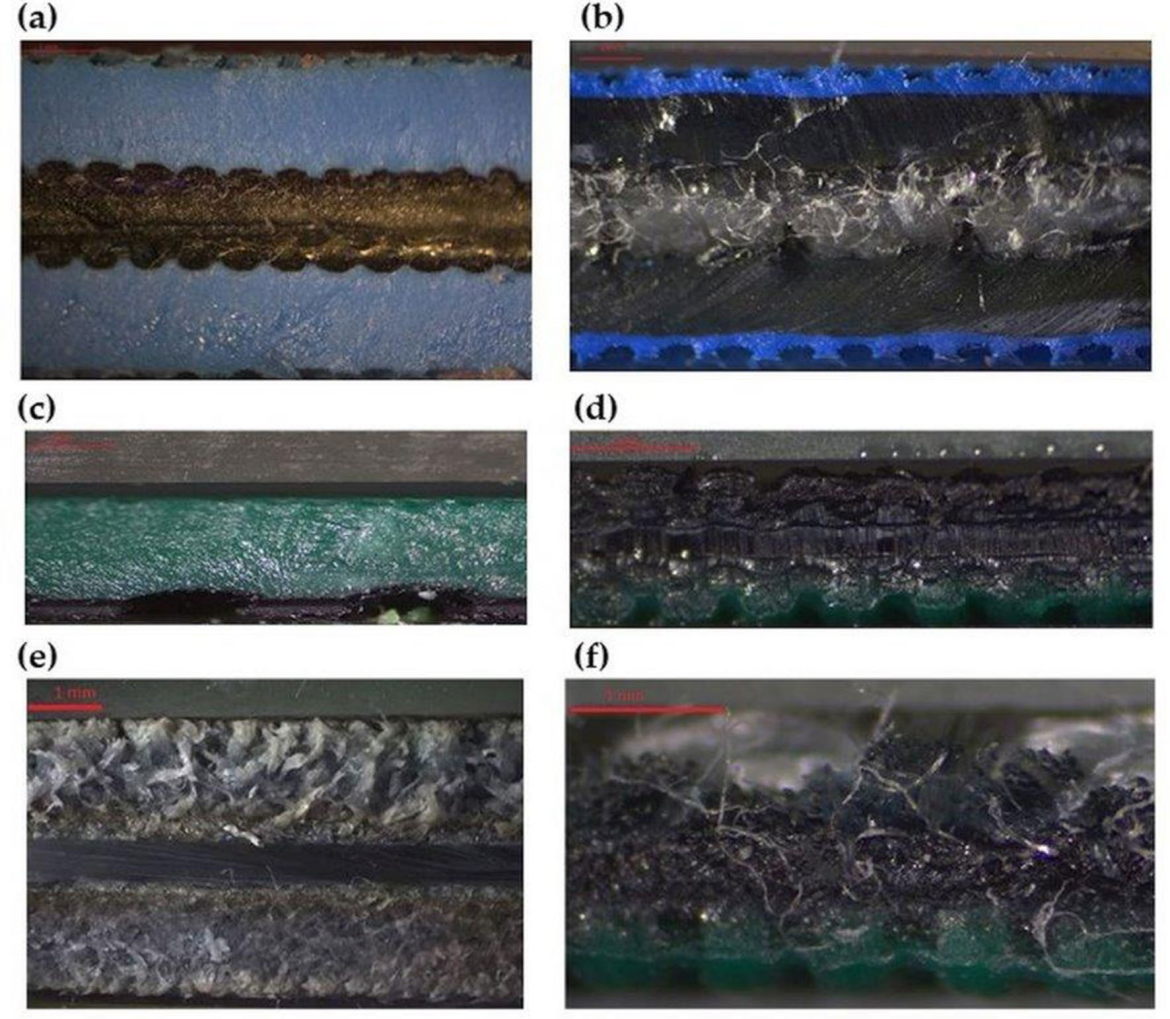
Optical light microscopy images of the internal structure of the flat belts: (**a**) XH, (**b**) TLA, (**c**) TC, (**d**) SG, (**e**) LL2, and (**f**) KSG.

The structures of all belts tested during operation with their markings are summarized in Table 1. In addition, Fig. 1 shows the morphology of the belt composites observed using optical light microscopy.

**Table 1:**
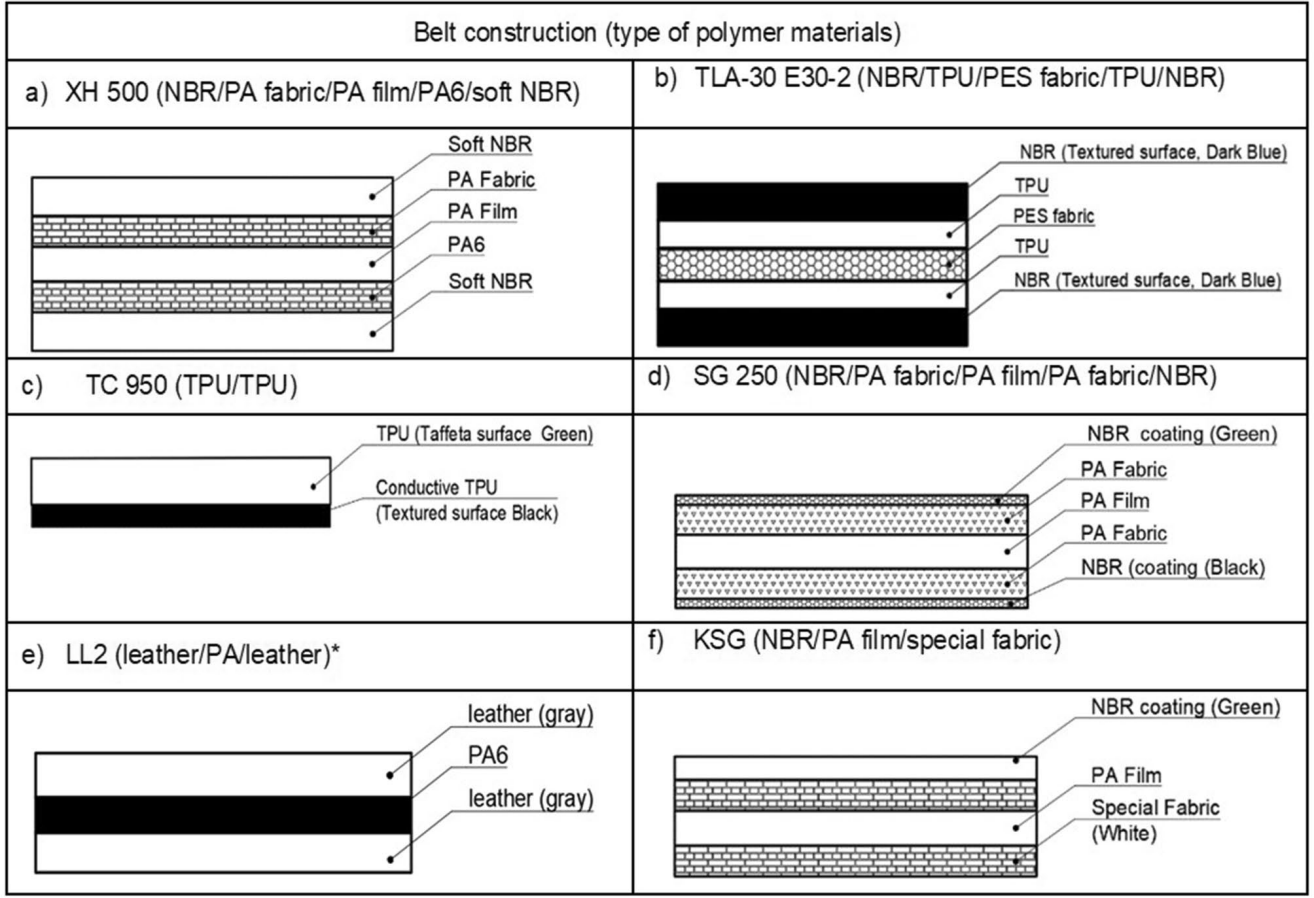
Markings and construction of the tested flat multilayer belts^29,30^.

The following symbols are used in Table 1: NBR—acrylonitrile butadiene rubber, PA fabric—polyamide fabric, PA film—polyamide film, PES—polyester cord, TPU—polyurethane, PA6—polyamide 6.

Table 1a lists the XH 500-4 belt type (*extra high top cower*)^29^, which consists of four layers. The top and bottom layers are acrylonitrile butadiene rubber (NBR), and the middle layers are polyamide film (PA) and polyamide fabric (PA_Fab_). This belt, similar to the previously described KSG belt, is characterized by high flexibility and excellent abrasion resistance and can work under conditions from -20 to 80 °C in printing houses.

Next, the TLA 30E 30 belt type (Table 1-b)^29^ was made of multilayer polymeric materials, i.e., the upper and bottom layers are acrylonitrile butadiene rubber (NBR), and the middle layers are made of polyurethane (TPU) and polyester fibre (PES). The TLA belt can be used in the tangential machine for textiles, where at operating temperatures ranging from 0 to 60 °C, it has a high abrasion resistance and a high friction coefficient and is capable of handling very heavy loads.

The TC 950 belt (*thermoplastic connection*) (Table 1-c)^29^ was made of polyurethane layers. The lower black layer has a rough structure, and the upper green layer has a smooth structure. The black surface is the running side of the belt, and the upper side can be used for transportation, e.g., in the textile industry. Such bands are used in drives characterized by a high speed of movement, and due to the construction, limited access to the belt. The operating temperature of the belt ranges from − 20 to 60 °C, its linear speed reaches 40 m/s, and because of considerable tensile stretch, it can be placed on pulleys without a tensioner. These belts are used in printing and textile industries in drives without the possibility of using pretension.

The SG 250 flat belt (Table 1-d)^29^ was made of several layers of NBR/PA fabric/PA film/NBR. It is characterized by easy assembly, a long service life, high flexibility, quiet running and an easy connection process. This belt can be used in the temperature range from − 20 to 80 °C; it shows high flexibility and optimal elongation during operation and can be used on small-diameter pulleys (from 35 mm). These belts are mainly used in printing, paper processing, packaging machine, parcel and letter sorting, and light transport applications.

The LL2 flat belt (*Leder Leder*)^30^ is made of three alternating layers of leather and polyamide 6 (PA6), as shown in Table 1-e. Such bands are used in multi-shaft drives in a contaminated working environment; they are characterized by a good resistance to variable loads; and they perform the function of overload couplings. These belts are characterized by a brief permanent slip, good cooperation with pulleys (the pulley does not damage the belt), and antistatic properties, and are designed to work in the temperature range from − 20 to + 100 °C. The closed belt was obtained by grinding its ends at an angle and heat sealing at 100–120 °C for 15 min. These types of belts are used in mills, chippers, machines and devices for wood processing.

Table 1-f shows that the KSG belt, which consists of three layers of NBR/PA film/special fabric and shows excellent abrasion resistance, high efficiency and flexibility, is long lasting and maintenance-free. At the same time, the band is characterized by a high resistance to oils, water and electrification while maintaining an operating temperature range from − 20 to 80 °C. The KSG belt is used in printing houses (folder gluers) in the production of packaging^29^.

### Characterization methods

In order to characterize the chemical decomposition of the belts, a JASCO FT/IR 4700 instrument (Tokyo, Japan) was used to obtain their Fourier transform infrared (FT-IR) spectra in the range from 400 to 4000 cm^−1^ with a resolution of 4 cm^−1^. Spectroscopic data were treated using the dedicated software Spectra Manager (ver. 2, JASCO, Easton, MD, US)^32^. The surface morphology of the flat belts was investigated by optical light microscopy (SK Opta-Tech) with an HDMI 6 OPTA-TECH RT 16 Mpx camera (OPTA-TECH, Warsaw, Poland) at 30 × magnification^32^.

### The thermal decomposition and combustion

**Figure 2:**
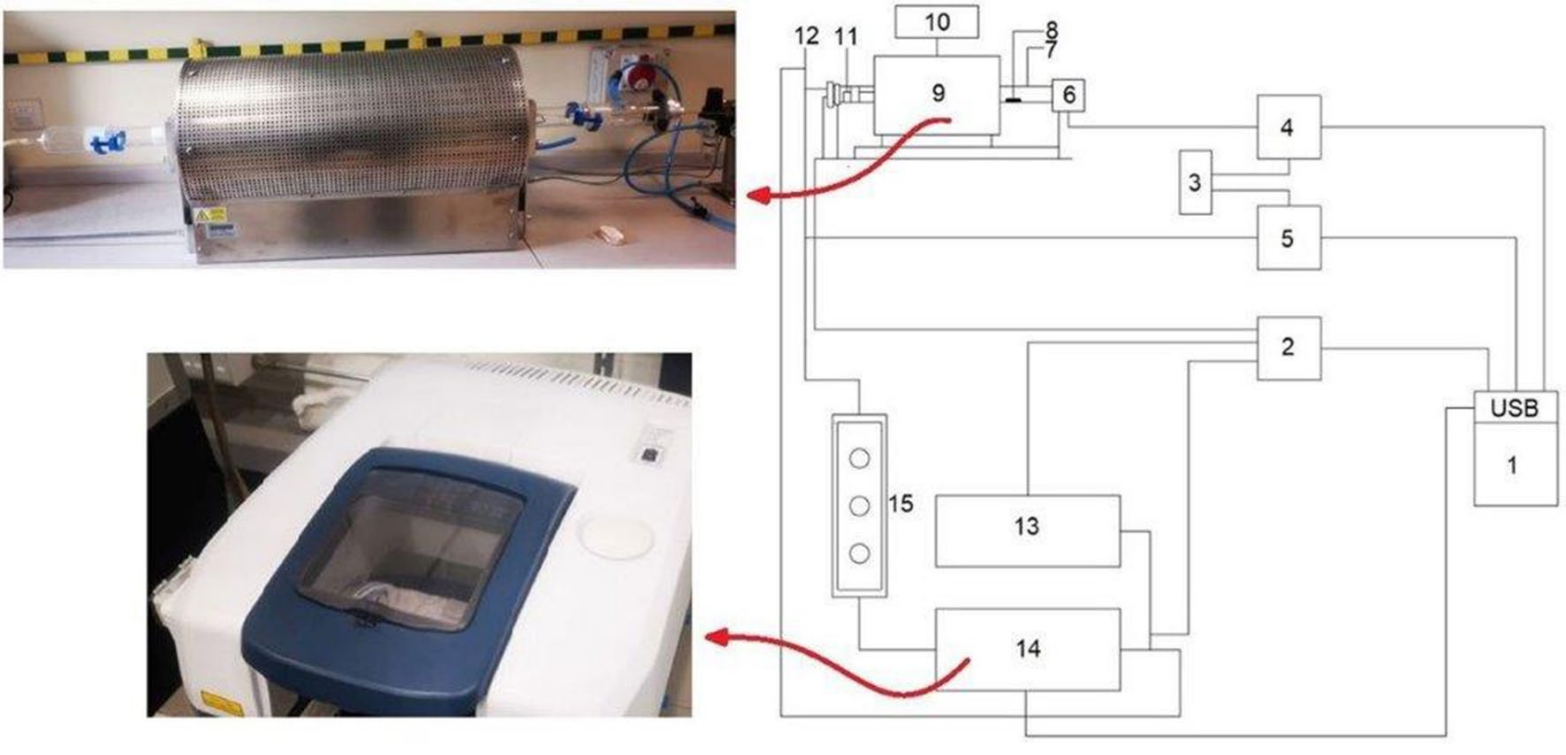
Stand for testing the toxicity of products of the combustion of materials used in the production of belts; 1—furnace, 2—FTIR gas analyser in Laboratory of Combustion and Explosion Processes of Józef Tuliszkowski Fire Protection Scientific and Research Center in Józefów).

The process of thermal decomposition and combustion of the XH, TLA, TC, SG, LL2, and KSG belts presented in Table 1 was carried out in stand testing, as presented in Fig. 2. Testing was conducted in a horizontal tube furnace at a temperature of 950 °C with air flow. The sample weights were 0.5 g, and the air flow was 26 l/h.

The tests were carried out in cooperation with the Complex of Laboratories of Combustion and Explosion Processes of the Fire Protection Science and Research Center of Józef Tuliszkowski in Józefów (Fig. 2). The research results will enrich the knowledge base for the industrial application of conveyor belts and drive belts.

Then, using a gas analyser, Fourier transform infrared (FTIR) spectroscopy coupled with a computer system was used to determine the mass of released substances (mass sample 0.5 g), and the specific emission and average volume concentration of selected chemical compounds were measured, i.e., carbon monoxide (CO), carbon dioxide (CO_2_), hydrogen cyanide (HCN), nitrogen dioxide (NO_2_), nitrogen oxide (NO), hydrogen chloride (HCl), sulfur dioxide (SO_2_), hydrogen bromide (HBr), and hydrogen fluoride (HF). Determining the specific emission of gases required the continuous measurement of the gas concentration as a function of time. ${LC}_{50}^{30}$ is an indicator of the limit concentration of the products of thermal decomposition and combustion.

For such measurements, an FT-IR gas analyser with a computer system was used to determine the mass released during the thermal decomposition and combustion of samples of the tested materials. The recorded values of the thermal decomposition and combustion products flowing through the control and measurement system were subjected to calculation algorithms depending on the sample mass and the gas volume flow rate. The specific emission of *Ex* measured gases was determined from Eq. (1).

1 $$Ex = \frac{{0.01 \cdot_{molx} \cdot T_{o} }}{{22.4 \cdot p_{o} }}$$

where ${M}_{molx}$ is the molecular weight of the measured component [g], ${p}_{o}$ is the pressure under normal conditions [Pa], and ${T}_{o}$ is the temperature under normal conditions [K].

## Results and discussion

**Figure 3:**
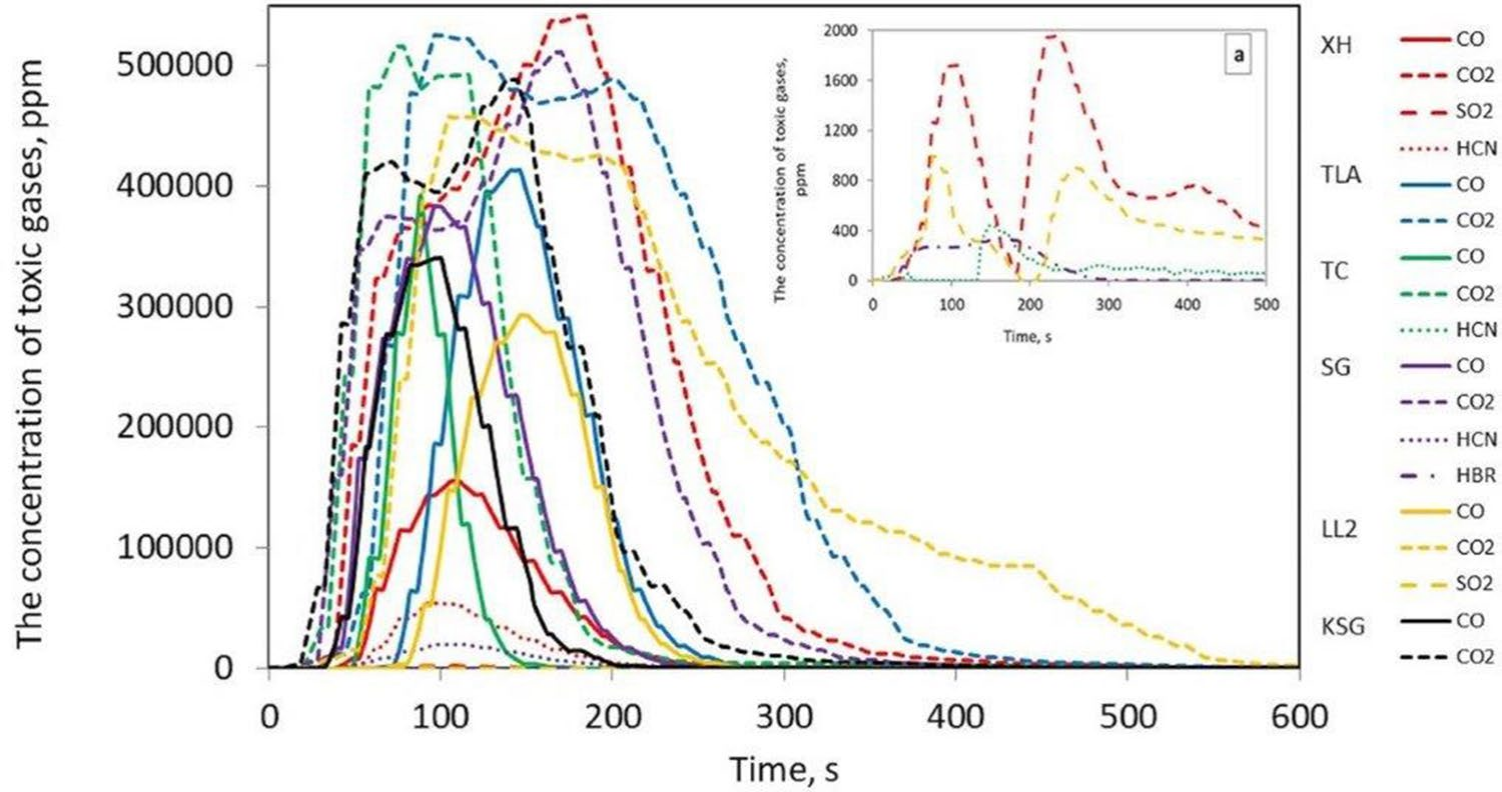
Toxic gas concentrations during the thermal decomposition and combustion of the flat drive belts; detail **a** shows a smaller measuring range from 0 to 2000 ppm.

As a result of the research, the concentrations of CO gases released during the thermal decomposition and combustion of six samples of conveyor belts or flat drive belts were determined and are illustrated as a function of time in Fig. 3.

Figure 3 shows that in the case of the tested belts, the emissions of 2 to 4 toxic compounds, such as CO, CO_2_, HCN, NO, SO_2_ and HBr, were recorded. It was found that the most frequently emitted gases during combustion were CO and CO_2_ for all tested belts, followed by HCN in 3 belts, such as XH-500, TC, SG 250, and SO_2_ in the case of the XH and LL2 belts. The emission of HBr compounds were only recorded in the SG 250 belt. Moreover, no emissions of NO_2_, NO, HCl, or HF compounds were recorded for the tested belts, as was the case with V-belts, the results of which are described in^10^. The number and type of toxins released during the burning of the belts are summarized in Table 2.

**Table 2:** The type and number of toxins emitted during the combustion of the tested samples.

Belt samples	Type of toxins emitted	The number of toxins emitted
XH 500-4	CO, CO_2_, SO_2_, HCN	4
TLA-30	CO, CO_2_	2
TC 950	CO, CO_2_, HCN	3
SG250	CO, CO_2_, HCN, HBr	4
LL2	CO, CO_2_, SO_2_,	3
KSG	CO, CO_2_	2

**Figure 4:**
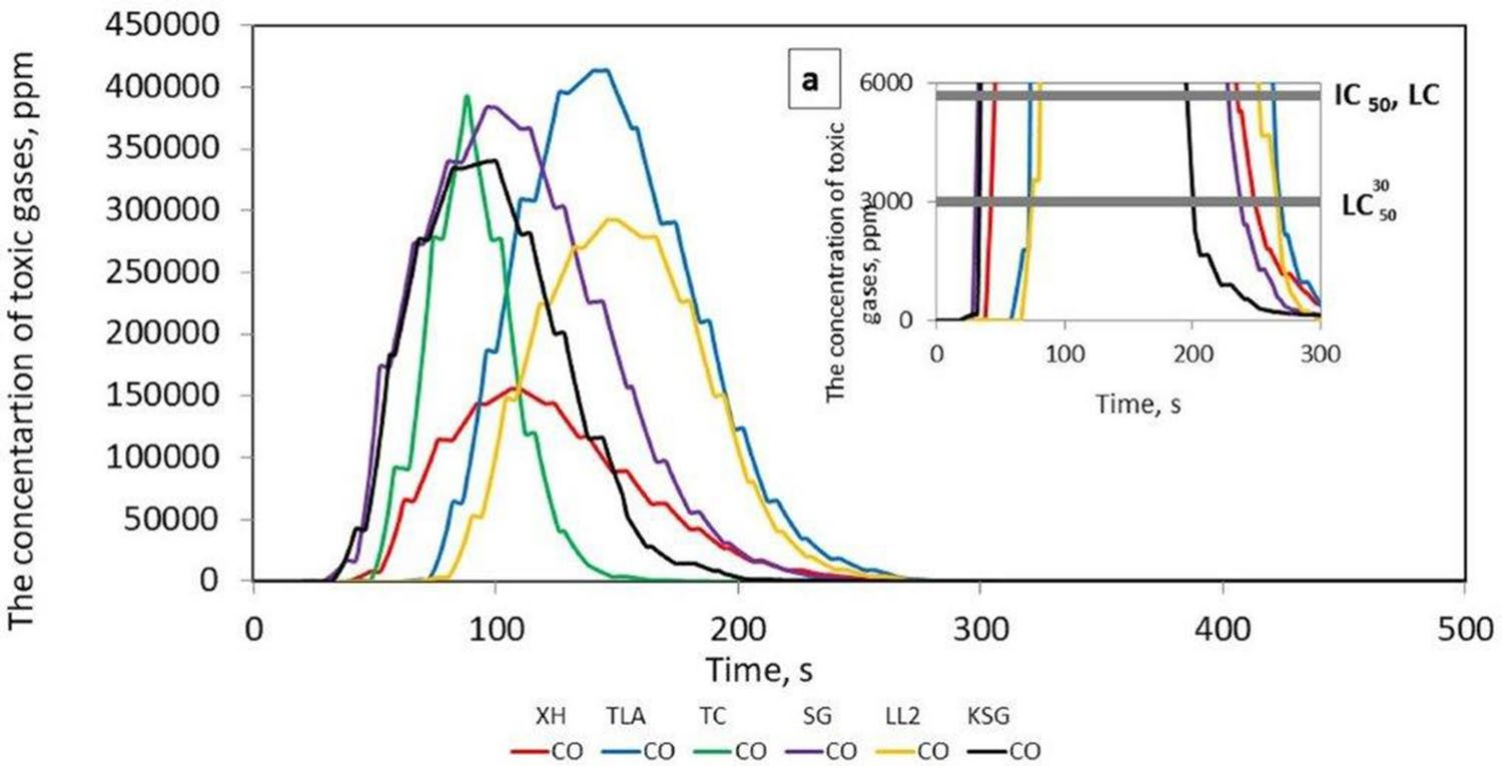
CO concentration as a function of time during thermal decomposition and combustion of the tested belt samples with permissible values (Table 2).

**Figure 5:**
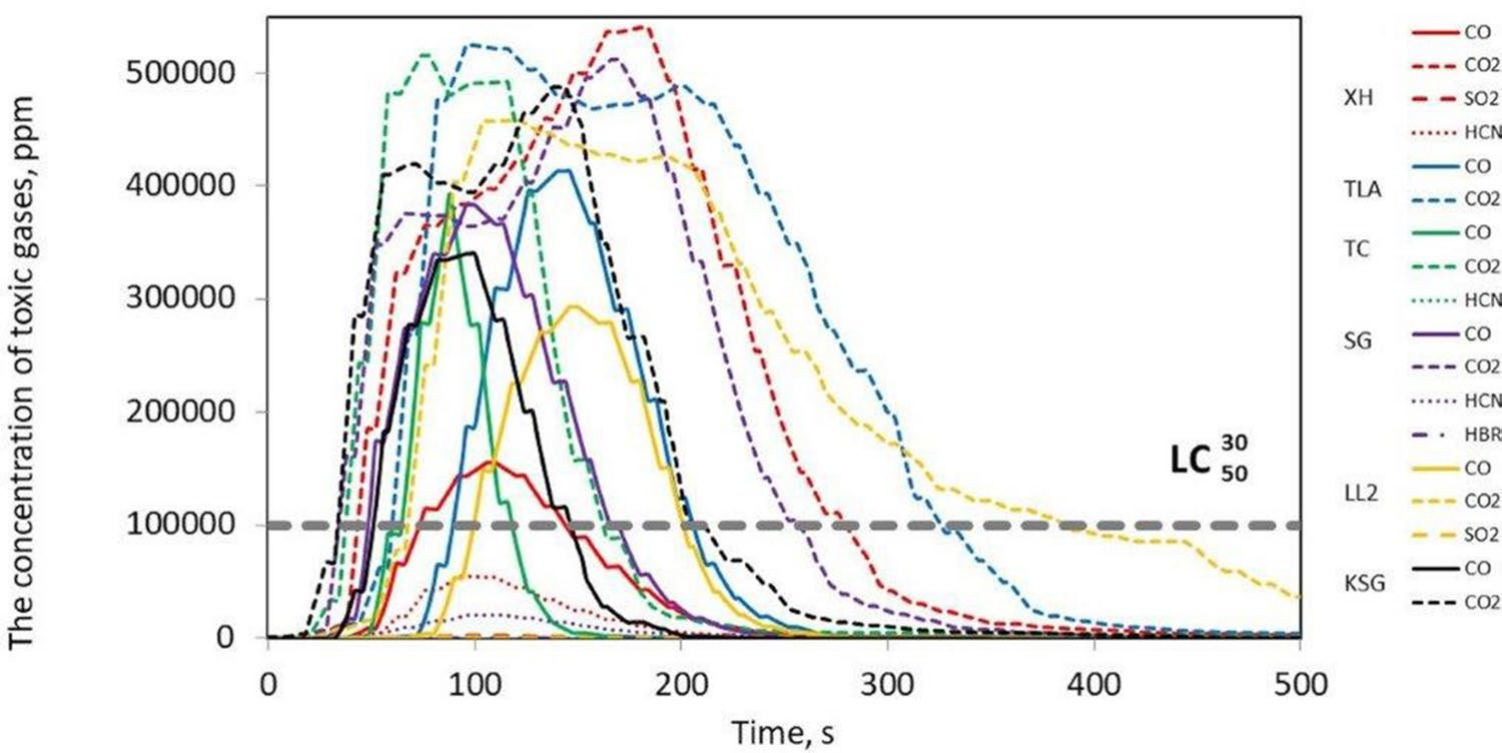
CO_2_ concentration as a function of time during thermal decomposition and combustion of the tested belt samples with the indication of permissible values (Table 2).

**Figure 6:**
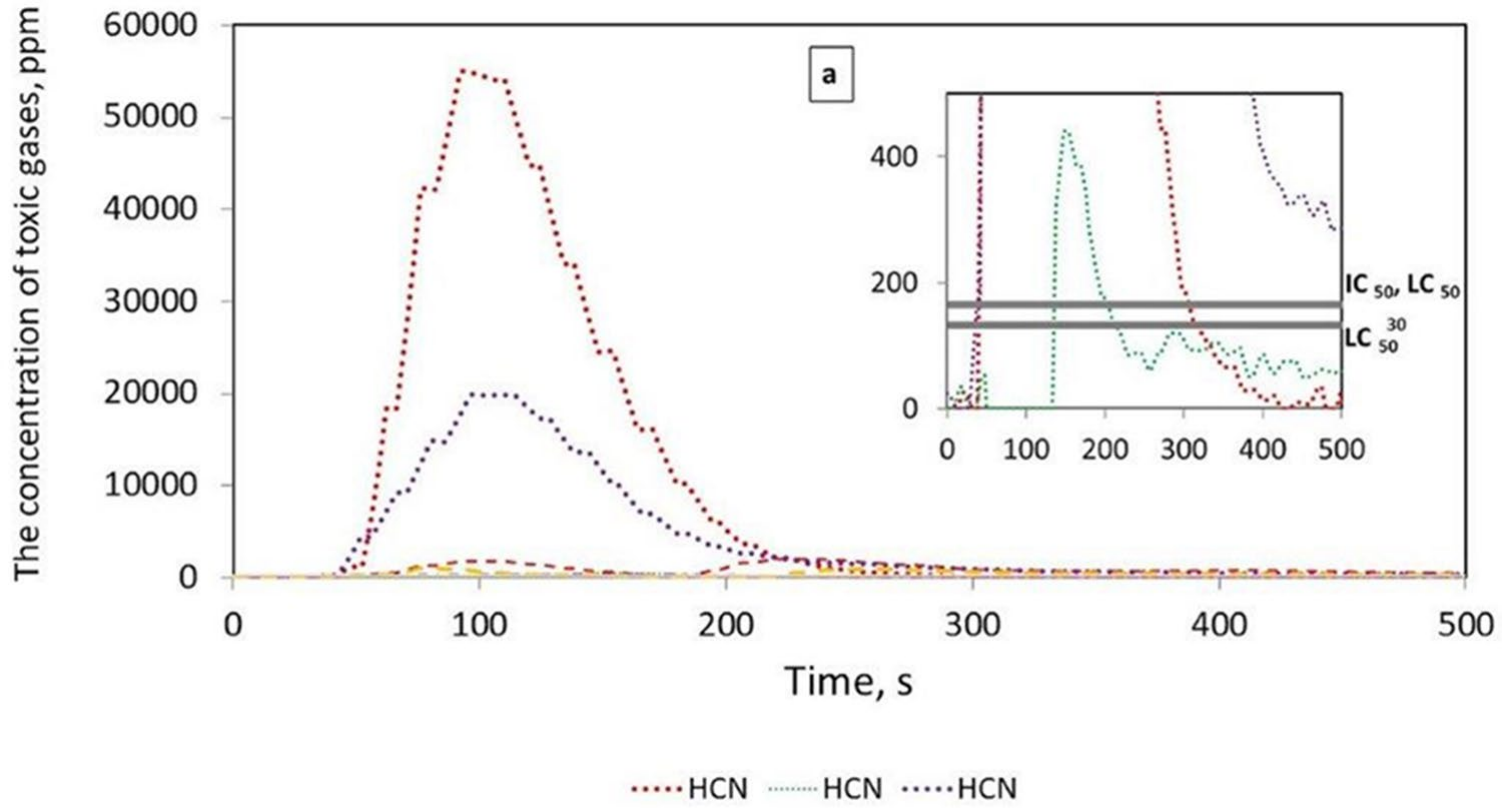
HCN concentration as a function of time during thermal decomposition and combustion of the tested belt samples with the indication of permissible values (Table 2).

**Figure 7:**
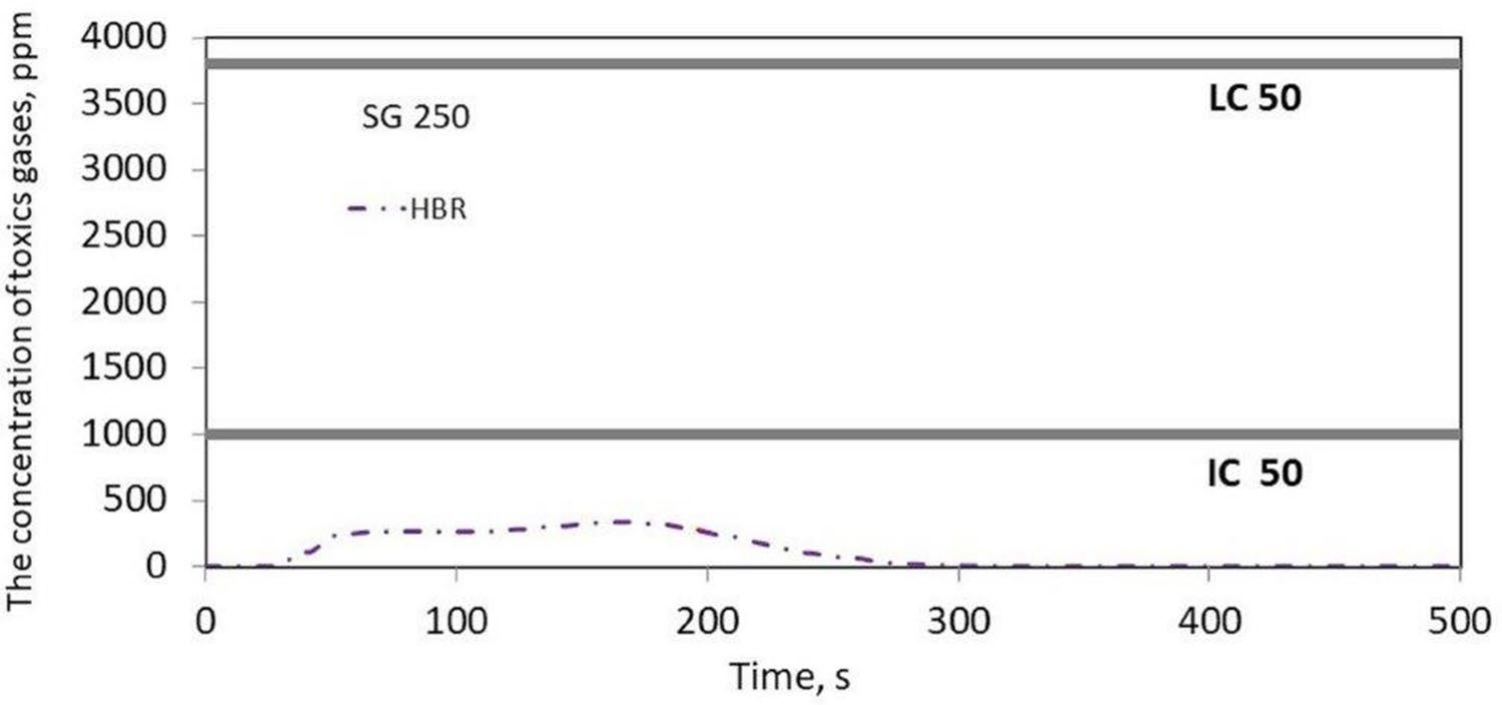
HBR concentration as a function of time during thermal decomposition and combustion of the tested belt samples with the indication of permissible values (Table 2).

**Figure 8:**
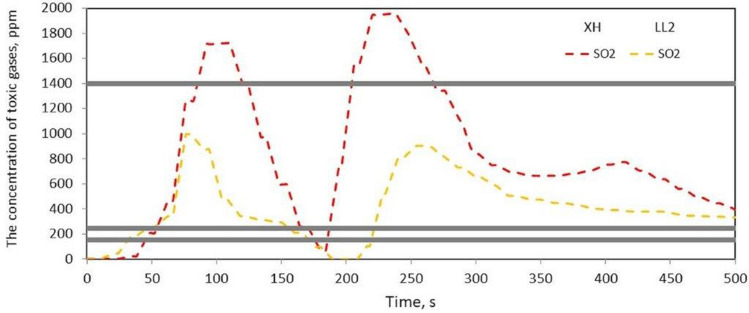
SO_2_ concentration as a function of time during thermal decomposition and combustion of the tested belt samples with the indication of permissible values (Table 2).

It should be stated that the XH 500-4 and SG 250 belts emit the most toxic substances into the atmosphere during combustion; hence, they pose a serious threat to human health and the environment. Figures 4, 5, 6, 7 and 8 show the instantaneous emission values of selected toxic gases depending on the type of belt material.

Additionally, Table 3 indicates the concentration limits of the products of the thermal decomposition and combustion of the tested materials.

**Table 3:** Concentration limits of the thermal decomposition products^21,31^.

Product of thermal decomposition	Concentration limits		
	Value $LC_{50i}^{30}$^21^	Value $IC_{50}$^31^	Value $LC_{50}$^31^
	(ppm)	(ppm)	(ppm)
Carbon monoxide (CO)	2999	5700	5700
Carbon dioxide (CO_2_)	99 963	It is not toxic but reduces the amount of O_2_ in the air, which causes hypoxia in the body	
Hydrogen cyanide (HCN)	133	165	165
Sulfur dioxide (SO_2_)	245	150	1400
Hydrogen bromide (HBr)	–	1000	3800

The *LC*_50_ parameter indicates the lethal concentration, i.e., the concentration of the substance at which 50% of the exposed organisms die during exposure or in a specified period after exposure^25^. The ${LC}_{50i}^{30}$ is the concentration that causes 50% of the population to die after 30 min of exposure, and *IC*_50_ is the inhibitory concentration that slows down the biological and biochemical functions of organisms by 50%^25^. Under real fire conditions, the mass of a burned belt is much greater than during the test, and the combustion process takes longer. For such conditions, the concentration limits for products of thermal decomposition and combustion of materials are used. The results showed that the CO (Fig. 4), CO_2_ (Fig. 5), and HCN (Fig. 6) emissions during the thermal decomposition and combustion of the tested samples significantly exceeded all permissible values.

Based on the research, it was found that the HBr emission (Fig. 7) did not exceed the permissible values, and in the case of SO_2_ emissions (Fig. 8), the permissible values ${LC}_{50i}^{30}$ and *IC*_50_ were exceeded during thermal decomposition and combustion of the samples of materials 1 and 5. However, the *LC*_50_ permissible value was only exceeded in the case of the SG belt.

Table 4 presents the characteristics of the emission of toxic compounds during the thermal decomposition and combustion of the tested samples under the analysed conditions.

**Table 4:**
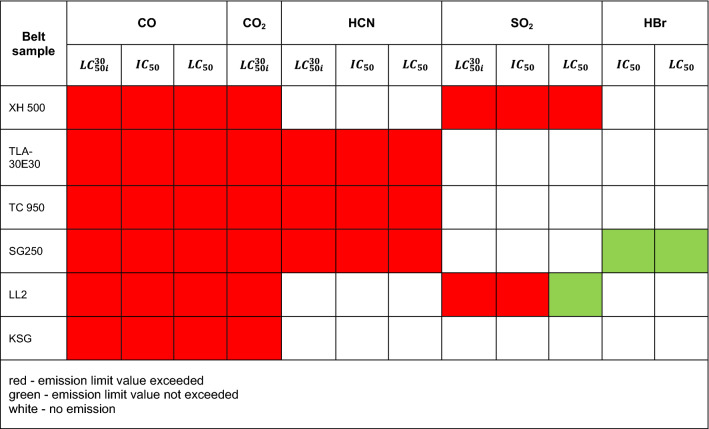
Characteristics of the emission of toxic compounds during thermal decomposition and combustion of the tested belts under the analysed conditions.

In addition, the results showed that during thermal decomposition and combustion of the samples of flat conveyor or drive belts at 950 °C, significant amounts of toxic chemicals (CO, CO_2_, HCN, HBr and SO_2_) were released.

It was found that in many belt cases, the concentrations of the emitted contaminants exceeding the ${LC}_{50i}^{30}$, *IC*_50_, and *LC*_50_ limit values were recorded within a few seconds of the process. To assess the behaviour of materials during a fire, a standard test methodology should be adopted^18^. Then, based on the products of thermal decomposition and combustion, it would be possible to characterize the materials used for the production of tension belts according to the toxicometric index as very toxic, toxic and moderately toxic under fire conditions. Parallel to the determination of the toxicity classes of tension belts, work should be carried out on limiting the emission of harmful compounds during their thermal decomposition, e.g., by using additives to reduce the flammability and toxicity of the materials used. Tests of tension belts conducted over the years have led to a significant improvement in their mechanical properties and resistance to operating conditions and environmental impact. However, it was not possible to significantly reduce the emission of contaminants during a fire. The characteristics of the concentration of gases emitted during thermal decomposition and combustion of belts made of various materials can serve as a database in systems monitoring the operation of machines and devices. The results showed that the flat drive belts exhibit less toxic properties under fire conditions than the V-belts.

**Figure 9:**
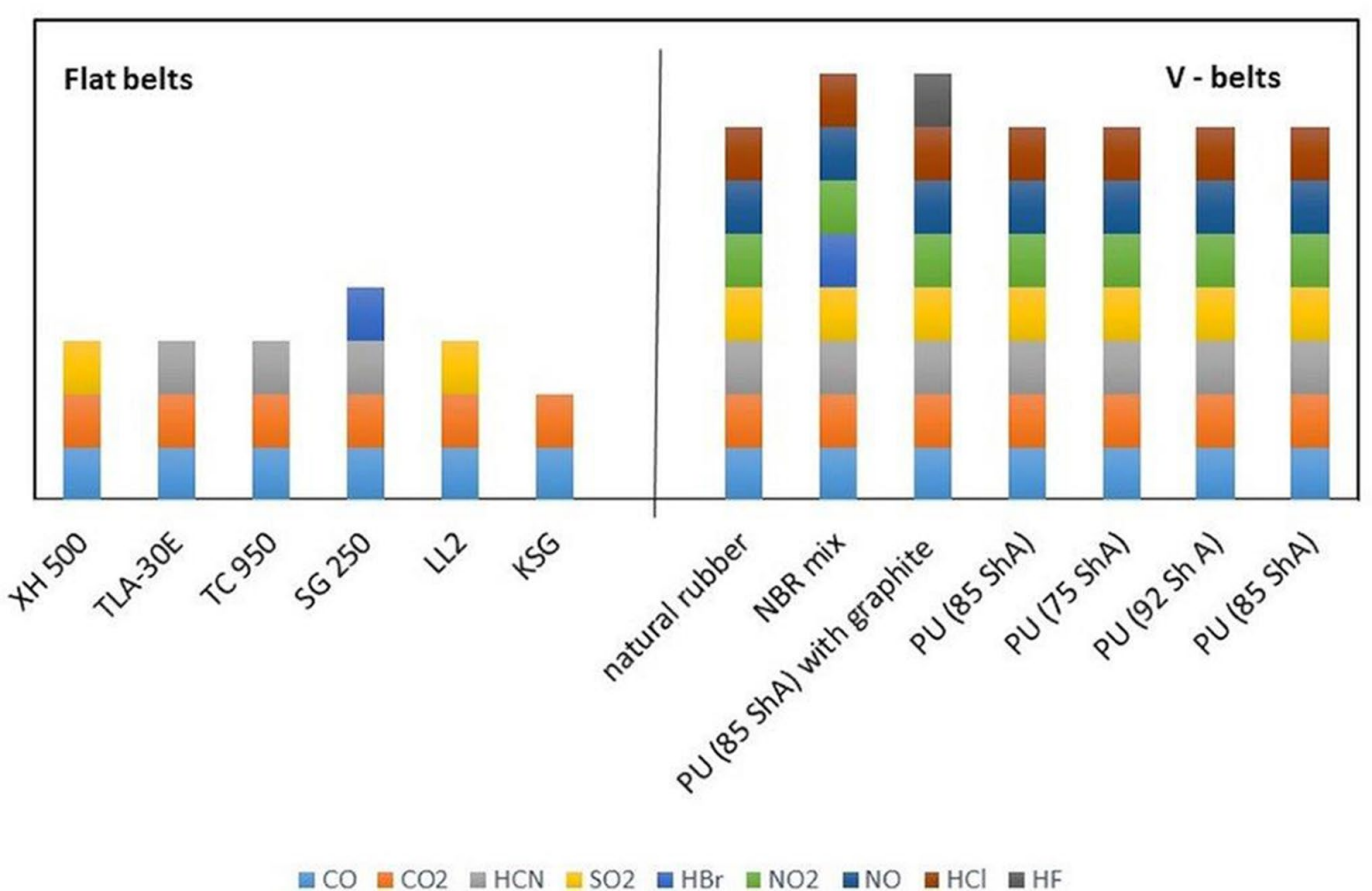
Qualitative comparison of the toxins emitted during thermal decomposition and combustion of flat belts and V-belts.

Figure 9 shows the qualitative comparison of toxins, such as CO, CO_2_, HCN, NO, NO_2_, HCl, SO_2_, HBr and HF, emitted during the thermal decomposition of combustion of flat belts and V-belts. This dependence was developed on the basis of the experimental tests of V-belts and flat belts. The results show that flat drive belts have less toxic properties under fire conditions than V-belts, mainly due to the kinds of materials, such as PA or NBR, with which the flat belts were made^10,32^. In paper^32^, the influence of various polymers, e.g., polyamide and polyurethane, on toxicity emissions was described. In the flat belt case, the dominant compounds released during combustion are CO and CO_2_ (all burned belts), as well as HCN, SO_2_ and HBr.

## Conclusions

Knowledge of the chemical composition and concentrations of chemical compounds emitted during the combustion of conveyor belts is important for the development of fire protection systems. Research teams developing conveyor fire protection systems are based on three basic types of measurement systems: temperature measurements, smoke measurements and air chemical composition measurements^33^. Integrated systems that use all three control methods are the modern trend in conveyor fire protection systems. The quick detection of the initial phase of fire is possible by using sensors along the route of the conveyor belt. In exemplary systems designed to control conveyor belts made of rubber belts, the detection systems are equipped with sensors of carbon monoxide (CO), hydrogen cyanide (HCN), smoke and temperature^34^ or are extended with sulfur dioxide (SO_2_) sensors^35^. Generally, the results show that classic conveyor belts (based on rubber) are characterized by the following emissions in the case of a fire: smoke, hydrocyanides (HCN), hydrochlorides (HCl), sulfur dioxide (SO_2_) and carbon monoxide CO^36^.

The results showed that under such conditions, modern conveyor belts may additionally emit carbon dioxide (CO_2_), sulfur dioxide (SO_2_) and hydrogen bromides (HBr). In the tested belts, HCl emissions were not noted. Multi-concentration gas detection to detect a fire is beneficial, especially in complex working conditions resulting from the type of transported product. Production areas can be contaminated with a variety of chemical emissions. For example, during the transport of coal and biomass, emissions of carbon monoxide (CO) and hydrocarbons (HC) are widespread^37,38^. This consequence is due to the phenomena occurring in these materials, e.g., gasification (CO and H_2_ emission), rot (CO emission) and fermentation (emission of H_2_, CH_4_ and complex hydrocarbons)^35^. The authors of this article propose to design detection systems adapted to recognize the emission of components that originate from the thermal decomposition and combustion of belts but not from production processes.
